# Supplementary material for: Operando visualisation of lithium plating by ultrasound imaging of battery cells
Source: Nat Commun. 2024 Nov 26;15:10237. doi: 10.1038/s41467-024-54319-6 (PMC11599900; doi:10.1038/s41467-024-54319-6)
Supplement: Supplementary file 1 — Supplementary Information [file 41467_2024_54319_MOESM1_ESM.pdf]

Operando Visualisation of Lithium Plating by Ultrasound Imaging of Battery Cells: **Supplementary Material**

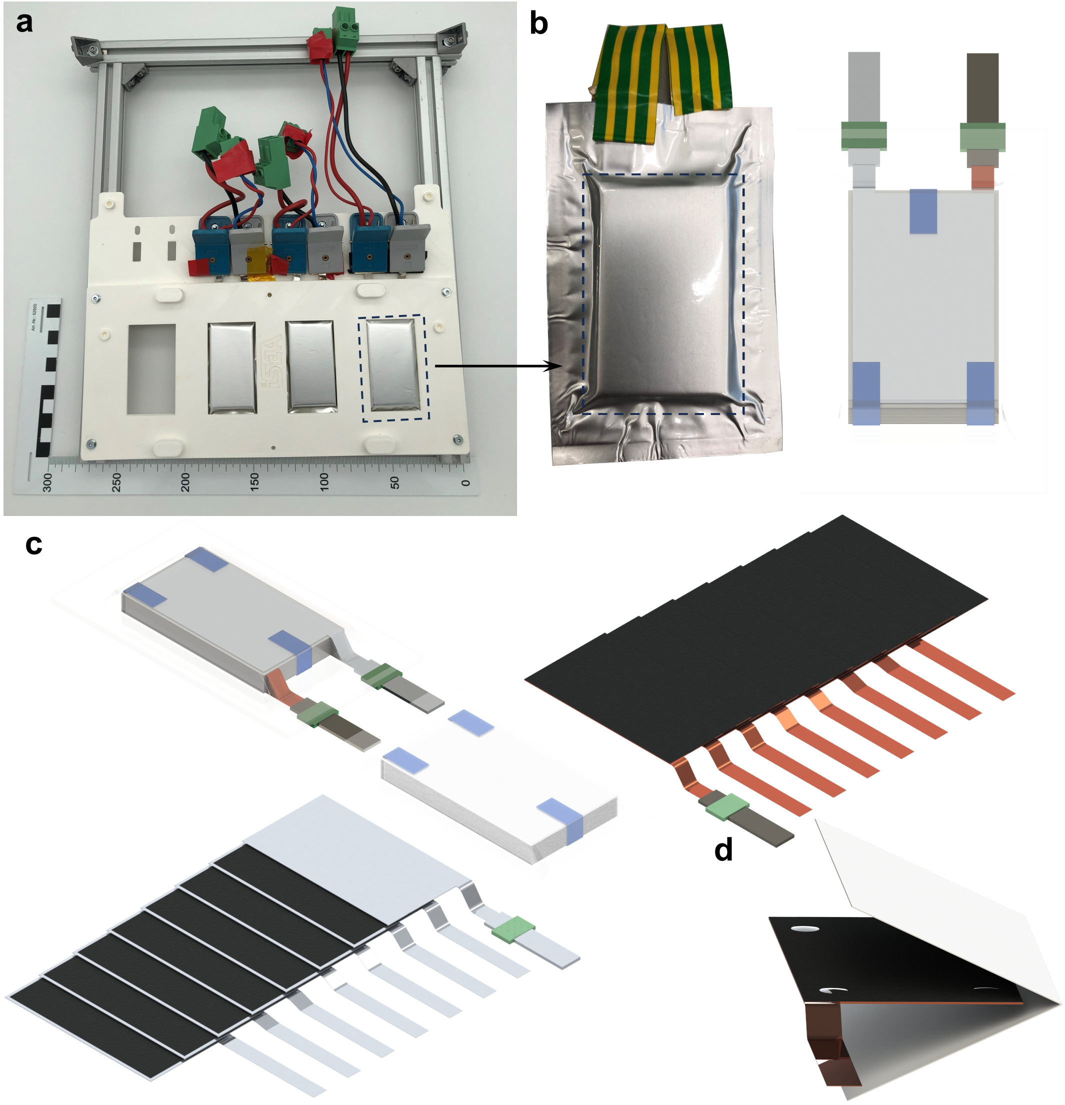

Supplementary Fig. 1: Specimen preparation for ultrasound imaging. **a** shows a photo of the cell holder which is fitted with 3 of 4 cells, analogous to Fig. 1. **b** shows a photo of the cells manufactured for these experiments, as well as a rendering without the pouch bag. **c** shows an isometric exploded view of the cell stack, as well as the assembled state. For better visibility, only seven out of 15 total layers are displayed. **d** shows how the adhesive dots were applied to the anode to limit the ion flow at these locations.

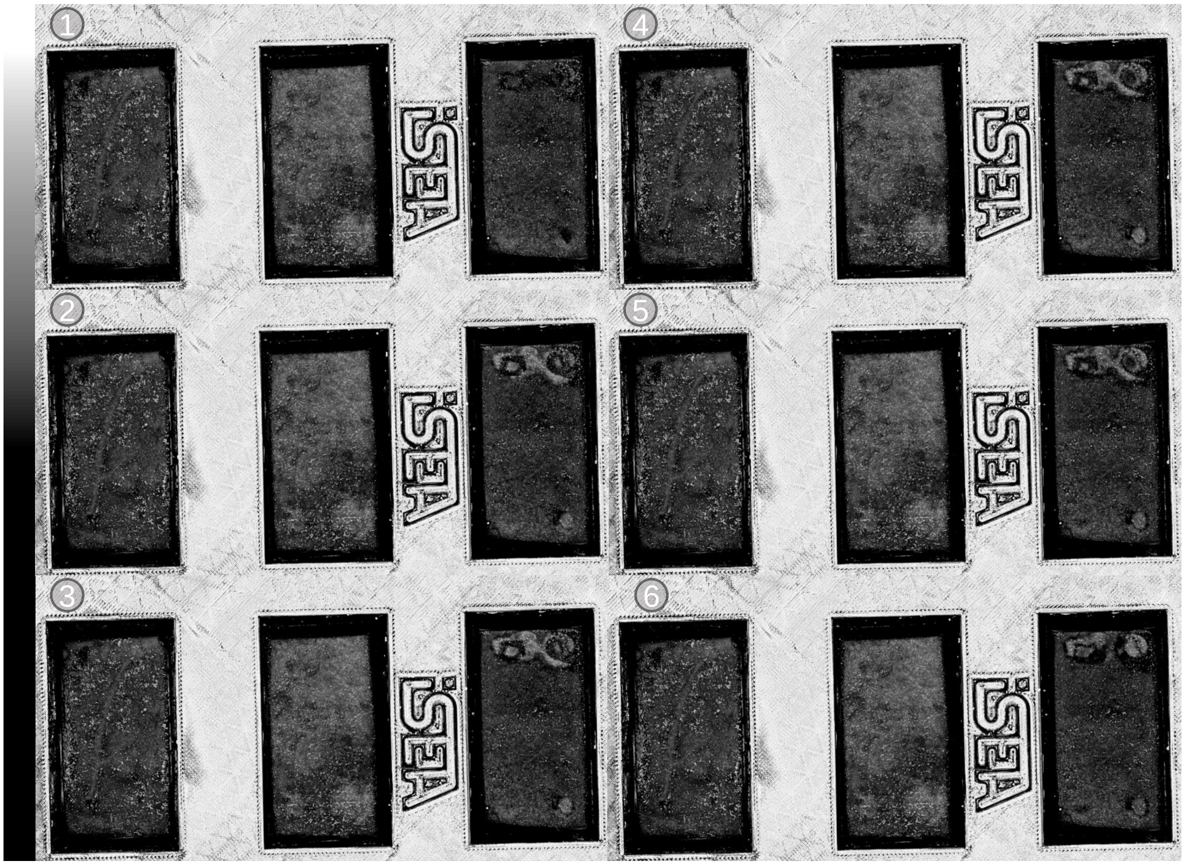

Supplementary Fig. 2: Ultrasound images of the cell holder with only the rightmost cell (cell 0) being charged. The right cell was charged with the identical protocol as in Fig. 2. The other two cells did not experience any electrochemical cycling. Since all three cells, as well as the cell holder, were scanned with an unnecessarily high resolution (10 µm), the scan time is 6 min. Nonetheless, similar indications as in Fig. 2 can be seen in state (2)-(6). State (1) marks the pristine state.

Material Parameters		
	Anode	Cathode
porosity	43.28%	43.34%
capacity density	$3.4\,\mathrm{mAh/cm^2}$	$3.0\,\mathrm{mAh/cm^2}$
avg. pore surface	$4.96\,\mathrm{m^2/g}$	$3.067\,\mathrm{m^2/g}$
avg. pore diameter	0.0863 µm	0.1599 µm
coating thickness	86 µm (Cu=18 µm)	90 µm (Al=20 µm)
electrode dimensions (width x height)	31 x 56 mm	29 x 55 mm
**Cell Parameters**		
capacity	1.4 Ah ± 0.06 Ah	
1 kHz resistance	40 mΩ	
voltage range	3.0 - 4.2 V	
self discharge	≤ 1.2 mV/d	
**Separator Parameters**		
name	Celgard 2325	
thickness	25 µm	
dimensions (width x height)	32.5 x 59 mm	

Supplementary Table 1: Material and cell parameters of the cell used in this work (manufactured by Fraunhofer ISIT, Germany).

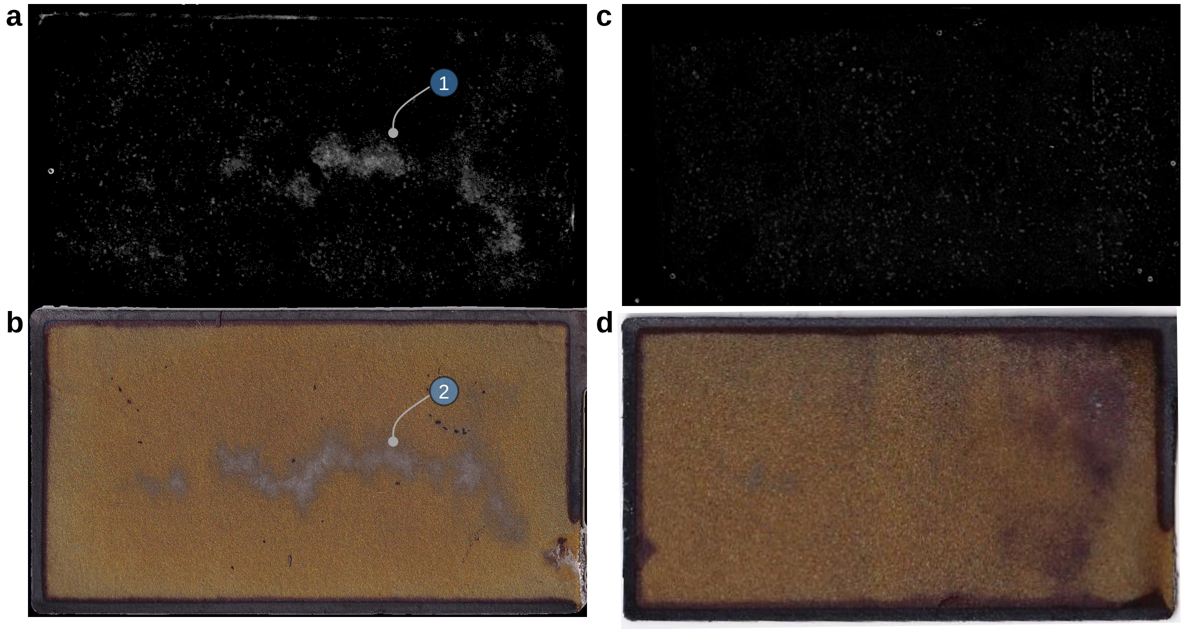

Supplementary Fig. 3: Detection of the gray area within battery cell 1 even before current is applied. **a** shows the ultrasound image under analysis of gate 4 instead of gate 3. This results in an analysis of the region, which is outside of the focal point. Therefore, the image has less detail and is blurry. Marking (1) indicates the gray area. **b** shows the corresponding anode image from the back of sheet 1. Here, marking (2) also indicates the gray deposits. Overall, this suggests that the gray deposits are already present before first charging cell 1. **c** represents the ultrasound image of cell 2 also using gate 4 instead of gate 3 before charging. **d** represents the corresponding optical image from the anode sheet under consideration. There are no grey depositions in the center of cell 2. This is confirmed by the ultrasound image in **c**.

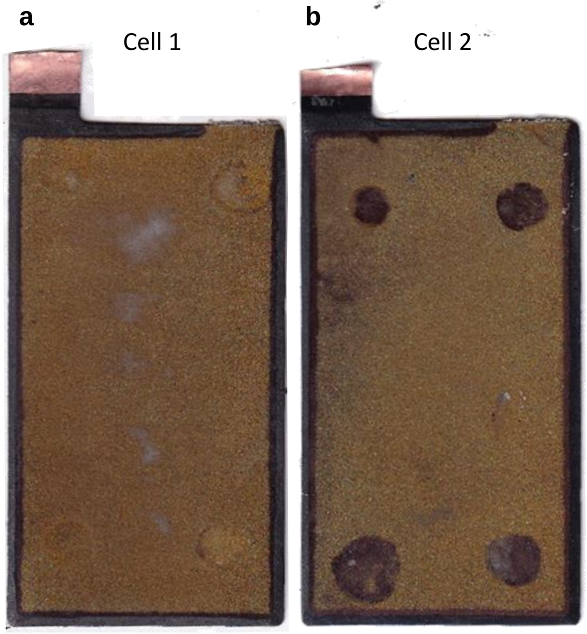

Supplementary Fig. 4: Comparison of the analyzed anode sheets from cell 1 and 2. a Represents an optical image of the front side of cell 1 and **b** of cell 2. It is clearly visible that the adhesive dots in cell 2 did not allow local lithiation of the anode (black hue). Given that the graphite beneath the adhesive dots on cell 1 exhibits a golden hue, it is apparent that lithiation is occurring in these areas. In conclusion, lithium plating has to develop around the adhesive dots of cell 2 instead of on top of them. This observation aligns with the findings from the operando ultrasound images presented in Fig. 2. In contrast to cell 1, cell 2 was idling for three weeks before disassembly, resulting in relaxation effects and altered color.

Explanation regarding the gray deposits in the center of cell 1

In the following, we discuss the formation of the gray deposits in the middle of the anode sheets of cell 1. Supplementary Fig. 4 shows optical images that clearly indicate that lithiation was possible under the adhesive dots of cell 1 and not of cell 2. In addition, no gray deposits are visible on cell 2 (see Supplementary Fig. 3 c and d). This suggests that the adhesive, which was mixed by hand and may have been exposed to air over time, had solidified more depending on the time of cell manufacturing for each individual cell. Due to the use of the initially liquid PVDF and acetone-based adhesive in the cells, it is hypothesized that parts of the still liquid adhesive or electrolyte-adhesive reaction products may have been forced into the central region of cell 1 during vacuum drawing. The adhesive or its reaction products likely accumulated beneath a separator cavity formed by partially solidified adhesive dots on the cell surface, leading to the deposition of fluorine in the center of the cell surface. Nevertheless, the utilization of the ultrasound imaging technique enables the differentiation and visualization of the plating behavior in both cell 1 and 2 and the appearance of the gray deposits.

Explanation regarding the increase in reflection amplitude upon charging

Literature investigating the transmission of ultrasonic waves through battery cells generally presents an increase in the transmitted wave amplitude when a battery cell is being charged [1, 2, 3]. Based on the examples by Hsieh et al., Davies et al. and Gold et al., this value is around 2-4% for commercial lithium-ion cells with a graphite anode. It appears, therefore, not immediately clear why the reflected amplitude also increases upon charging. With constant attenuation (diffraction, scattering, and absorption) and constant injected amplitude (normally ensured by the pulse generator, coupling agent, and transducer),

an increase in the reflected amplitude would reduce the transmitted amplitude. However, since several publications in the literature have observed an increase in the reflected amplitude upon charging, we assume that a reduction in the attenuation in the battery cell leads to an increase in the transmitted amplitude. Reduced attenuation would increase the amplitude of both the reflected and transmitted waves. Our findings, in combination with existing literature, suggest that this is the dominant effect causing the reflection amplitude to rise upon charging [2].

Adhesive pattern

The PVDF and acetone-based adhesive dots were placed in a semi-alternating pattern to ensure a homogeneous electrode stack despite local inhomogeneities. These adhesive dots, as shown in supplementary Fig. 5, were applied only to the anodes and not to the cathodes. While their primary role in this work is to trigger lithium plating, these adhesive dots are typically used to enhance bonding between the electrode and separator in commercial battery cell manufacturing [4]. This helps in aligning the battery cell components during assembly. Based on this, additional adhesive dots were added to the four corners of the outermost anodes.

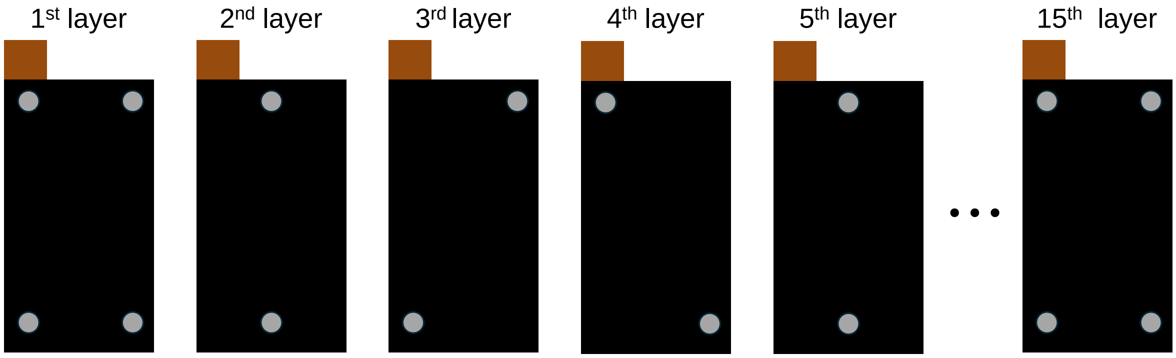

Supplementary Fig. 5: Adhesive placement pattern on the anode sheets. Alternating the positioning of the adhesive dots leads to a more homogeneous geometric shape of the electrode stack, as this prevents two adhesive dots from lying on top of each other.

Possible ultrasound detection depth

To fully demonstrate the possible detection depth using the small-wavelength transducers (25 MHz frequency, 66 µm wavelength in cells), we performed the imaging method described in chapter 4.3 again using different transducer-cell distances. By doing so, we effectively control the penetration depth of the focal zone within the battery cell. This allows the visualization throughout different depths within the cell. Since no cells were available for the investigation after the preliminary tests and primary measurement series, a cell from the manufacturer LiFun of comparable size was used. The format and size of the selected cell are commonly employed in cell development [5, 6]. Specifically, we use the LiFun cell with model number 5575166 for our investigation. The cell has an NMC811 cathode and a C+20 %SiO anode. Similar to the Fraunhofer cell, the LiFun cell has a capacity of 1 Ah and a thickness of 4 mm (9 anodes and 8 cathodes, double-sided coated). The cell was filled with 3.4 ml of a $LiPF_6$ in EC:EMC 3:7 based electrolyte (manufactured by E-Lyte Innovations GmbH, Germany) and then formed and degassed according to the manufacturer's specifications.

A circular indentation with a diameter of 10 mm was carefully imprinted on the back of the battery cell to induce a mechanical feature as far away from the transducer as possible. This is shown in Supplementary Fig. 6a. Additionally, another smaller indentation was made roughly in the middle of the cell's backside. However, due to a congruent defect on the front side, this indentation was not further investigated and thus omitted. The front side of the cell features typical manufacturer bar

codes and labels, as well as scratches and deformations of the pouch bag caused by Kapton tape on the electrode stack. Moreover, the cell's surface shows additional scratches and dents from handling after manufacturing, as well as remnants of internal tracking stickers. The objective is to move the sensor closer to the battery cell and capture ultrasonic images at fixed intervals until the indentation appears on the ultrasound image.

The results of the ultrasonic measurements are shown in Supplementary Fig. 6b. The first row shows the ultrasound image of the battery cell's surface. For this measurement, the sensor was positioned precisely at the focal length specified in the datasheet (1.905 cm in this case). In the ultrasound image, the cell's barcode, the deformation of the pouch bag by the Kapton tape, and the dents and scratches are clearly visible. Additionally, some black spots are visible due to small air bubbles in the freshly tapped distilled water that had not yet dissipated. Next, the sensor was moved to a distance of 1.885 cm, similar to the procedure described in the main manuscript. The corresponding ultrasound image now shows clear measurements of the Kapton tape with minimal surface effects. Only the dents on the surface, which deflect the incident ultrasound angle, leave artifacts on the ultrasound images. Apart from that, the ultrasound image of the electrode appears homogeneous. Subsequently, the sensor was moved further by 4 mm, approximately the thickness of the cell, resulting in a distance of 1.505 cm from the cell. The corresponding ultrasound image still shows artifacts from the dents on the surface. Additionally, the ultrasound image is relatively inhomogeneous, which could be due to the number of interactions at all interfaces. Nevertheless, the circular indentation on the side of the cell is visible. However, unlike the effects seen on the upper layers, the circular indentation is not as sharply defined. This may be due to interactions outside the focal point becoming comparable in magnitude to those within the focal point along an 8mm test path at this depth (4 mm in and 4 mm out). Future research should investigate whether interactions outside the focal point can be filtered out through appropriate signal processing.

In summary, it has been demonstrated that detection of effects through an approximately 4 mm thick cell is achievable with the selected sensors. It has been observed that signal quality diminishes and ultrasound images become less homogeneous with deeper layers despite focusing. For thicker cells or stringent detection requirements in lower layers, adapting the measurement hardware may be advisable. This could include increasing excitation amplitude, reducing ultrasound frequency (thus minimizing attenuation), among other adjustments. Balancing all parameters according to the cell characteristics and detection requirements should therefore always be prioritized.

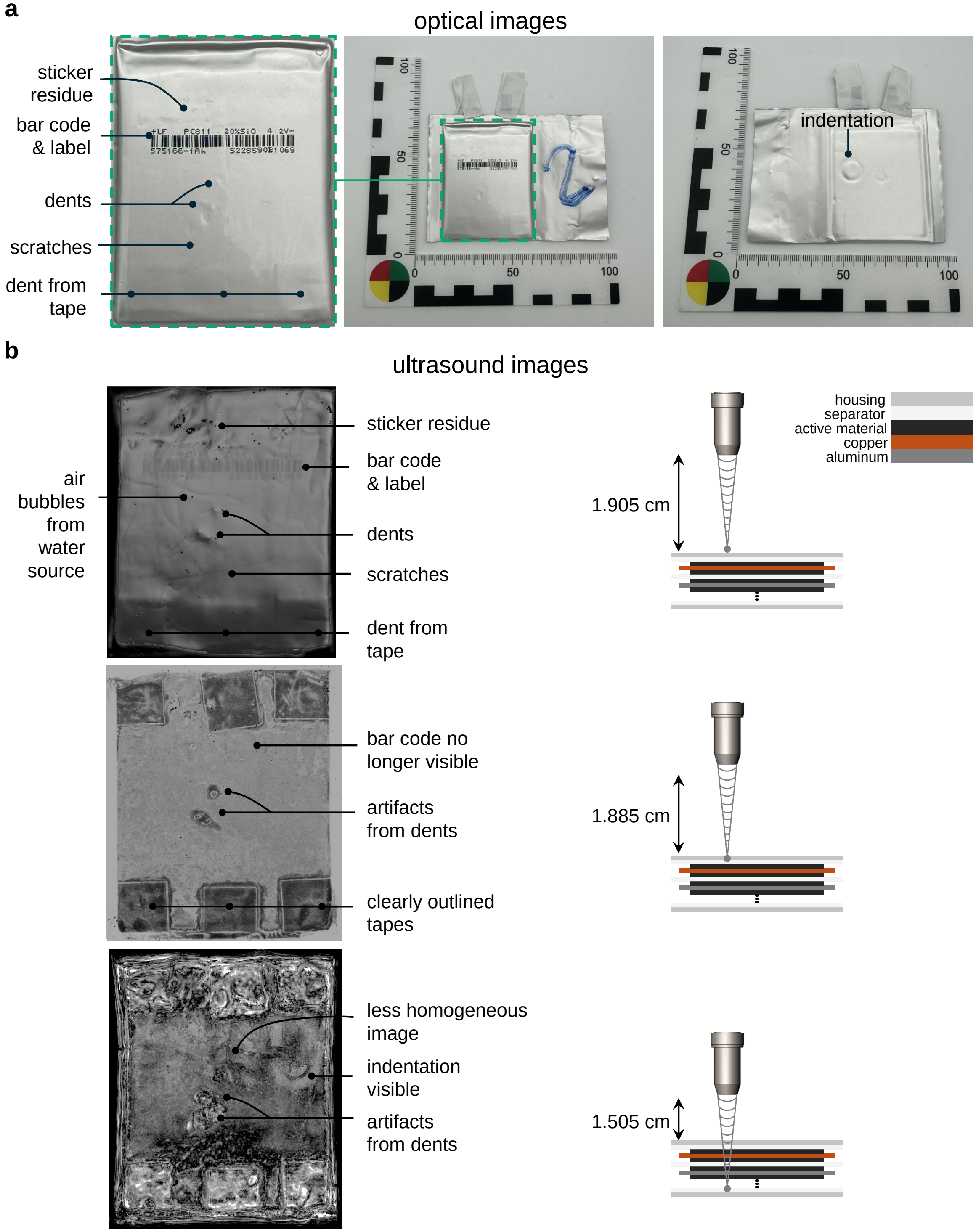

Supplementary Fig. 6: Demonstration of the possible penetration depth using 25 MHz **transducers a** shows optical images of the sample LiFun 5575166. **b** shows the corresponding ultrasound images of the sample at different sensor distances, which produces images at different depths within the cell. It is possible to visualize mechanical features on the other side of the cell with the same sensors. However, the image sharpness at greater depths could be increased by using lower sound frequencies.

Alternative failure modes

Given that most battery ultrasound imaging research focuses on electrolyte distribution, dry out, or gas generation, we will explain in this chapter why we are convinced that the indications in Fig. 2 are due to lithium deposits rather than these common failure modes.

1. Electrolyte dry out: Since we see a contraction of the bright spots in the ultrasound images at the beginning of the CV phase, during relaxation after charging and when the cell is being discharged, and a ring-like expansion upon subsequent charging, and electrolyte dry out is a non-reversible process, it can be assumed that this effect did not play a significant role in our investigations.

2. Gas formation: Similar to electrolyte dry out, gas generation is also a non-reversible process in most cases. However, it could be argued that if the current rate is reduced, the gas generation could stop, and the gas could partially migrate into cavities that are not on the surface of the electrode stacks (for example on the side of the electrode stack). To have a direct comparison to ultrasound images with provoked gas generation, calendar aging tests were carried out in a separate study. Here, gas generation was specifically provoked in a Fraunhofer cell. The corresponding ultrasound image can be seen in supplementary Fig. 7 a. In contrast to the ultrasound images in Fig. 2 of the manuscript, the gas regions in the ultrasound image of supplementary Fig. 7 a are smooth and homogeneous, making it impossible to detect the electrode structure beneath. Additionally, the gas would need to be generated precisely on the lithium deposits and migrate laterally, as it would remain in the focal zone during vertical migration into the electrode. An argument against this occurring in the cells shown in Fig. 2 is that ultrasound images of cell 2, taken over several hours after charging without electrochemical cycling, show no drifts to or from the bright spots.
   In a separate study, a different pouch cell was not fully degassed. Due to incorrectly set parameters in the vacuum regulator and an insufficiently deep-drawn pouch casing, only the gas in the center of the cell surface was removed, leaving some gas at the edge of the cell. The corresponding ultrasound image with a different color map can be seen in supplementary Fig. 7 b. It is again clearly recognizable that there is no information about the structural details of the electrodes in the gas areas and that there is a sharp edge to the non-gassed areas. Once again, the ultrasound image at the gas points is smooth and homogeneous in contrast to the spots in Fig. 2. Alternatively, to the verification steps in the revised manuscript, such ultrasound examinations could be performed in parallel to operando neutron imaging in future studies to obtain additional verification of the results [7].

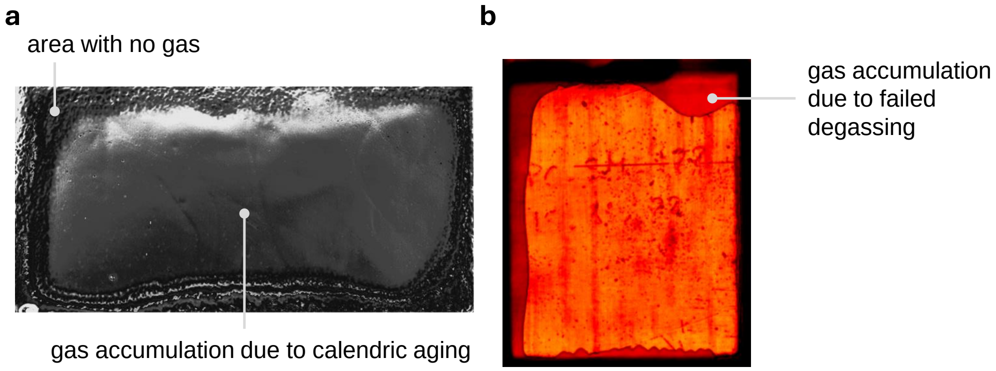

Supplementary Fig. 7: Ultrasound images of gassing in different pouch cells. The images were created using the same settings and transducer as described in section 4.3. **a** shows the ultrasound image of a cell in which gas genaration has been provoked by calendar aging with the gas accumulating in the center of the cell surface. **b** shows another cell in which gas has accumulated at the edge of the cell due to failed degassing and insufficient deep drawing of the housing. In both cases, the gas is outlined by a clear line, causes a homogeneous reflection and does not allow interpretation of the electrode structure under the gas.

Supplementary References

[1] G. Davies, K. W. Knehr, B. V. Tassell, T. Hodson, S. Biswas, A. G. Hsieh, and D. A. Steingart, "State of charge and state of health estimation using electrochemical acoustic time of flight analysis," *Journal of The Electrochemical Society*, vol. 164, p. A2746, sep 2017.

[2] A. G. Hsieh, S. Bhadra, B. J. Hertzberg, P. J. Gjeltema, A. Goy, J. W. Fleischer, and D. A. Steingart, "Electrochemical-acoustic time of flight: in operando correlation of physical dynamics with battery charge and health," *Energy Environ. Sci.*, vol. 8, pp. 1569–1577, 2015.

[3] L. Gold, T. Bach, W. Virsik, A. Schmitt, J. Müller, T. E. Staab, and G. Sextl, "Probing lithium-ion batteries' state-of-charge using ultrasonic transmission – concept and laboratory testing," *Journal of Power Sources*, vol. 343, pp. 536–544, 2017.

[4] G. Huang, H. Wu, G. Cao, Z. Liu, H. Hu, and S. Guo, "Application of a new polymer particle adhesive for lithium battery separators," *Coatings*, vol. 13, no. 1, 2023.

[5] F. Dai and M. Cai, "Best practices in lithium battery cell preparation and evaluation," *Communications Materials*, vol. 3, p. 64, Sep 2022.

[6] V. Murray, D. S. Hall, and J. R. Dahn, "A guide to full coin cell making for academic researchers," *Journal of The Electrochemical Society*, vol. 166, p. A329, jan 2019.

[7] B. Michalak, H. Sommer, D. Mannes, A. Kaestner, T. Brezesinski, and J. Janek, "Gas evolution in operating lithium-ion batteries studied in situ by neutron imaging," *Scientific Reports*, vol. 5, p. 15627, Oct 2015.
