# Supplementary material for: Operando visualisation of lithium plating by ultrasound imaging of battery cells
Source: Nat Commun. 2024 Nov 26;15:10237. doi: 10.1038/s41467-024-54319-6 (PMC11599900; doi:10.1038/s41467-024-54319-6)
Supplement: Supplementary file 2 — Description of Additional Supplementary Files [file 41467_2024_54319_MOESM2_ESM.docx]

**Description of Additional Supplementary Information**

Supplementary Movie 1: Ultrasound image sequencing of cell 1. Bright indications become clearly visible during charging. The dynamics of these indications are further illustrated in Fig. 2a.

Supplementary Movie 2: Ultrasound image sequence of cell 2. Bright spots emerge during charging, shrink during discharge, and enlarge again in the following charge cycle. The detailed dynamics of these spots are presented in Fig. 2c.
